# Can Training Enhance Face Cognition Abilities in Middle-Aged Adults?

**DOI:** 10.1371/journal.pone.0090249

**Published:** 2014-03-14

**Authors:** Dominika Dolzycka, Grit Herzmann, Werner Sommer, Oliver Wilhelm

**Affiliations:** 1 Department of Psychology, Humboldt-Universitaet zu Berlin, Berlin, Germany; 2 Department of Psychology, The College of Wooster, Wooster, Ohio, United States of America; 3 Department of Psychology, University Ulm, Ulm, Germany; Tel Aviv University, Israel

## Abstract

Face cognition is a crucial skill for social interaction and shows large individual differences in healthy adults, suggesting a possibility for improvement in some. We developed and tested specific training procedures for the accuracy of face memory and the speed of face cognition. Two groups each of 20 healthy middle-aged trainees practiced for 29 daily sessions of 15 minutes duration with different computerized home-based training procedures. In addition, 20 matched and 59 non-matched controls were included. Face cognition speed training enhanced performance during the training and transferred to the latent factor level as measured in a pre-post comparison. Persistence of the training effect was evidenced at the manifest level after three months. However, the training procedure influenced the speed of processing object stimuli to the same extent as face stimuli and therefore seems to have affected a more general ability of processing complex visual stimuli and not only faces. No effects of training on the accuracy of face memory were found. This study demonstrates that face-specific abilities may be hard to improve but also shows the plasticity of the speed of processing complex visual stimuli – for the first time in middle-aged, normal adults.

## Introduction

Face cognition is essential for successful social interactions and many professions require face cognition skills. Recently, research on individual differences has indicated that though all adults are highly experienced in face cognition there are large interindividual variations in this skill [1]–[6]. While some individuals are extremely good at recognizing faces they have seen before [7], [8], others range at the lower end of this distribution [9], [10]. Intervention procedures have been helpful in enhancing social functioning in impaired individuals, like facial emotion recognition in patients with autism or with schizophrenia [11]–[13]. A training of face cognition might be of advantage also for unimpaired individuals who wish to improve their face cognition for personal or professional reasons. Finally, experimental training studies on face cognition can contribute to the understanding of the psychological processes underlying this skill. It is the scope of this study to develop and test two training procedures aiming at the improvement of specific face cognition abilities.

### Models of Face Cognition

The starting point for the present study was the three-factor model of face cognition developed by Wilhelm et al. [5], which was based on the classic cognitive model of Bruce and Young [14]. The latter model specifies seven cognitive processing stages for face recognition. First, structural features are extracted and composed into a viewer-centered primary sketch. From here, expression, facial speech, and directed visual processing are analyzed in parallel, whereas face recognition proceeds in sequence. For face recognition, the percept is compared to representations of faces stored in long-term memory, namely in face recognition units. If the percept matches a representation and the face recognition unit is sufficiently triggered, then further semantic information can be activated in units termed person identity nodes. Although the independence of the parallel processes has sometimes been questioned (e.g., [15]–[17]), the Bruce and Young model is still widely considered to capture the essence of available experimental findings.

Whereas the Bruce and Young model is based on findings from experimental and clinical studies, a complimentary approach to the structure of cognitive processes is to study individual differences. Using a multivariate approach Wilhelm et al. [5] established a three factor model of face cognition consisting of the ability factors face perception, face memory, and speed of face cognition. Face perception is the ability to perceive facial features and their configuration accurately. It is measured with several indicators (that is, tasks) requiring perceptual comparisons devoid of memory load. Face memory is the ability to recognize learned faces accurately. It is measured with tasks that involve memorizing faces and their subsequent recognition. Speed of face cognition encompasses the swiftness of perceiving, learning, and recognizing unfamiliar faces. Therefore, indicators of this ability require perceptual comparisons and recognition of faces, but the tasks are so easy that unimpaired adults differ primarily in their response latencies. Face perception and face memory are highly correlated but separable, whereas both abilities are only weakly related to the ability of speed of face cognition. These three abilities constitute face-specific skills that were shown to be distinct from the established ability constructs of immediate and delayed memory, mental speed, object cognition, and general cognitive ability [5]. Hildebrandt et al. [18] replicated the three-factor model and showed that face cognition ability remains invariant over the age range from 18 to 88 years. This three-factor model of face cognition was the basis for the training study reported in the present paper. In this study, two training procedures were developed which aimed at improving the abilities of face memory and the speed of face cognition. We focused on these two factors because attempts to train face perception have already been made [19]–[22].

### Requirements for Design and Evaluation of Training Studies

Summarizing the literature, a training intervention should aim to improve a skill in a sustainable and lasting manner. Support for such effects requires a design with at least three measurement time points: pre-test, post-test directly after the training, and at least one follow-up measurement [23]. The results should be compared to those of a control group, which received a treatment that did not influence the factors the training aimed at but otherwise was as similar to the training treatment as possible [24], [25]. Near-transfer and far-transfer effects should be assessed [26].

### Previous Attempts to Train Face Cognition

We focus here only on findings concerning own-race face cognition. Other-race face recognition training is a different field of research. Therefore, the results concerning such findings are not reported. Studies on training face cognition in healthy adults date back to the 70 s and 80 s. These investigations aimed at contrasting the outcomes of different general training procedures, but they were not effective or even had negative effects. For example, Malpass [27] trained different groups in feature analysis, global personality judgment, global facial judgment, or repeated face recognition tests in 12 one-hour sessions. However, the training degraded face cognition for all groups. Likewise, Woodhead, Baddeley, and Simmonds [28] found no reliable gains after three training sessions in either memorizing or categorizing faces. In two other studies, the recognition of faces from other ethnic groups was trained for 1.5 or 4 hours [29], [30]. Training improved recognition for faces from the trained ethnic group, but it did not increase performance for faces from the own ethnic group. Malpass, Lavigueur, and Weldon [31] reported two experiments. In Experiment 1, they combined different durations of training (2, 4, or 8 hours) with three different verbal training strategies (describing faces, recognizing faces from descriptions, or describing differences between triads of faces) and found that none was effective on visual face recognition. In Experiment 2, training lasted less than an hour and combined practice on faces of a certain ethnic group (own or other) with different feedback methods (no feedback, verbal feedback, electric shock feedback). For faces from the own ethnic group, they found a decrease in performance. Sporer [32] compared different encoding strategies and could show that deeper encoding strategies (scanning the whole face) were superior to mere feature-based strategies but performance did not exceed that of a no-instruction control group. A study found that general, unspecific practice did not increase the ability of face cognition [33]. Three experiments compared the performance of identity verification for novices, passport inspectors, and police officers of a specialist investigating task force for upright as well as for inverted photographs. Performance was highly error-prone, was further reduced by inversion, and most interestingly did not differ between security personnel and novices.

Two main reasons for the ineffectiveness of the training studies reviewed above were suggested. First, the trainees were already at their ceiling performance of face cognition due to the extensive everyday experience they have had with faces [27]–[30], [32]. Second, the costs of switching from the normally used to the experimentally required face-recognition strategy might have counteracted possible training-related increases [31], [34], [35]. All of the above studies aimed at the general ability of face cognition and interventions were of short duration. Since participants arguably had developed their own strategies for recognizing and remembering faces developed in everyday life situations these short interventions might have led them to abandon their strategies and replace them with insufficiently trained new ones [35]–[39]. Third, as Malpass [27] argues, insufficient understanding of the processes underlying face cognition might have hindered the development of effective intervention programs and thus resulted in not finding the expected effects.

The reviewed studies indicate that mere exposure or the repeated act of identifying faces does not suffice to enhance average face cognition ability. Newer training studies were more specific and often aimed at particular abilities as recommended in the recent training literature [40], [41]. However, most of these newer training studies concentrated on impaired face cognition abilities. For example several studies showed that patients with Alzheimer's dementia succeeded in learning of face-name pairs through everyday practice [42]–[44], as did patients with cognitive deficits [45]. In case studies, with prosopagnosic individuals expert-like performance was achieved for Greebles [46], [47] or for other objects classes [48]. Taken together, these studies demonstrate that persons with selective deficits may benefit from extensive and specific training.

One prerequisite of recognition is perception. There are a few recent studies that specifically investigated training of face perception, one of the abilities of face cognition. Training identification of either upright or inverted faces strongly increased performance on the trained identities and the trained view [49]. These results generalized only marginally to new faces and to the untrained orientation hinting at specificity of perceptual learning. Though, general training of face cognition did not improve performance for persons with prosopagnosia [46], [47], [50], several case studies reported positive effects of specific face perception training [19], [20], [22]. For example, over three months of training on discriminating faces by their spatial configuration improved face identification to the level of healthy controls [22]. Two experiments [21] investigated the plasticity of face perception in persons without face cognition deficits. In one study, the effects of participation in a portrait-painting course were analyzed and, in the other, the effects of training perceiving differences between morphs of faces. Deliberate practice influenced performance in both studies as intended, but the effects were small.

To summarize, the studies reviewed above indicate that specific training procedures for participants with deficits, expertise training with face-like objects, as well as specific training of face perception improved performance as intended. As any memory contents, training improvements deteriorate with time if the training is not refreshed [37]. One shortcoming of the results reported above is that training effects were measured directly after the interventions at the time point when the largest improvements were to be expected.

### Training Abilities of Face Cognition

There are substantial individual differences in the ability of face cognition in healthy adults. Possibly due to the discouraging results of initial attempts, training this important social ability has been neglected in recent research. Though some of the above-mentioned studies investigated training of face perception none was designed to directly address the question of training other abilities of face cognition. Therefore, the present study investigated training effects in face memory and speed of face cognition in a healthy middle-aged population. This approach is based on the premise that training cognitive component abilities can enhance the ability itself [37], [51]. Also, an effective training might be interesting for people with occupations requiring good face cognition abilities. A large-scale internet-based study of face memory, with over 60.000 participants, found that performance on this ability peaks in the early thirties [52]. Hildebrandt et al. [18] showed that age-related decreases in the ability of speed of face processing begin in the thirties and of face memory – especially in men – in the forties, whereas the ability of face perception is preserved until the sixties. Thus, development of an effective training for the two abilities that start to decay earlier might bring a remedy for persons still engaged in occupations requiring face cognition.

This work is the first attempt to specifically train face memory and speed of face cognition. The memory training task is based on the model of Bruce and Young [14], where known faces are encoded as face recognition units. These codes are robust over time and independent of perspective. In our training procedure, faces had to be learned and recognized later. To ensure that training duration was constant across participants and comparable to the speed training duration, the difficulty level was adapted by changing memory demands (i.e., similarity between faces). Further, an additional non-learned perspective was used in the test blocks to assess if perspective-independent codes had been generated.

The component ability of speed of face cognition has been recently distinguished as a separate ability factor in latent factor models by Wilhelm et al. [5]. Speed of face cognition is conceptualized as generalizing over both face perception and face memory. The speed training tasks were constructed as very easy tasks to which all participants can respond correctly if given enough time. In such tasks, the speed of responding is the differentiating variable between individuals. In the present training, one task demanded fast perceptual processing and the other had a minimal memory load and required fast memory processing. The difficulty level was adapted by individually changing the reaction time deadline while keeping performance consistently high.

The aims of this study were to develop and test two training regimes that enhanced face cognition by improving performance on its factor abilities. It was hypothesized that the abilities aimed at will improve (effectiveness aim) and that other abilities will not be influenced (discriminant validity aim). Effectiveness of training was tested at several levels. First, during training there should be a strong increase of the task-specific performance. Second, performance on non-trained tasks measuring the relevant ability should be enhanced at post-test showing effectiveness at the manifest level. Third, modeling of the post-test data should display better performance on the trained abilities at the latent factor level. Discriminant validity of training effects was to be established [37] by assessing whether each training procedure influenced only the ability it aimed for while leaving other abilities unaffected. For example, the speed of face cognition training regime should not impact performance on face perception, face memory, object cognition, or general cognitive ability measures.

### Overview of Study

The present study re-recruited a subsample of participants investigated by Hildebrandt et al. [18]; the original psychometric data from this study served as pre-test. The study was designed according to the above delineated recommendations and requirements for training interventions. Two computerized training procedures were developed. Participants trained at home on adaptive tasks for approximately 15 minutes per day for 29 days. Post-tests were conducted a few days after the end of the training phase and after an interval of another three months. The training effects were assessed with a wide range of tasks. Besides tasks measuring performance on face and object cognition, further indicators for far-transfer were included, i.e. for immediate and delayed memory, general cognitive ability, and mental speed.

## Methods

### Participants

Participants were recruited from the study conducted by Hildebrandt et al. [18]. The authors had used a battery of tasks with several indicators for each ability of face cognition, object cognition, and other cognitive abilities (for task descriptions see [53]). Hence, it was possible to match the groups for this study on a number of parameters. Sixty middle-aged subjects, who consented to participate in the training study, were assigned to one of three matched groups. Fifty-nine further participants were recruited from the above-mentioned sample as unmatched control group. This group was needed to obtain a sample size adequate for calculating structural equation models. The three matched groups participated in the pre-test and in the first and the second post-tests, whereas the unmatched controls participated in the pre-test and first post-test only.

For matching, triads of persons with similar factor scores on all three abilities of face cognition were created. These persons were randomly assigned to one of the two training groups or to the matched control group. The three matched groups did not differ in initial factor scores on face memory, face perception, speed of face processing, general cognition, immediate and delayed memory, and mental speed, nor in age or gender (for details see Table 1).

**Table 1:** Sample Means and Standard Deviations for Practice and Control Groups.

	Practice groups		Control groups		*p* *	*f* *
	Memory	Speed	Matched	Unmatched		
*N*	19 (9 men)	20 (10 men)	20 (13 men)	59 (24 men)		
Age range	28–58	27–57	27–60	17–70		
Mean Age	44.8 (8.3)	42.7 (8.8)	43.1 (11.4)	46.1(18.2)	.76	.10
FS face perception	.39 (.73)	.29 (.73)	.40 (.64)	.18 (.88)	.62	.14
FS face memory	.44 (.67)	.31 (.77)	.36 (.81)	.20 (.89)	.69	.10
FS face speed	.24 (.65)	.25 (.61)	.21 (.87)	.12 (.92)	.91	.10
FS general cognition	.10 (.17)	.05 (.17)	.04 (.25)	.03 (.23)	.65	.18
FS immediate and delayed memory	.63 (.08)	.66 (.10)	.62 (.10)	.66 (.08)	.26	.03
FS mental speed	1.05 (.11)	1.07 (.13)	1.07 (.10)	1.05 (.11)	.79	.10

Note. FS: factor score; SDs are in parenthesis.

* p-value and effect size f for the comparison of three matched groups (memory, speed, and matched control).

The study was approved by the institutional ethics committee of the Humboldt-University, Vote 2013-39, and conducted in accordance with the Declaration of Helsinki. All participants took part in the study voluntarily. At the beginning of the study, participants received comprehensive verbal and written information about the procedure and purpose of the study. Then they were free to decide whether or not they wanted to participate. Subjects were paid for their participation and could withdraw from the study at any time without any penalty.

Initially, each matched group comprised 20 participants. During the training period, one participant dropped out of the memory group. Each trainee was paid 88 EUR plus an additional 6 to 24 EUR based on performance. Each participant of the matched and unmatched control groups received 45 and 21 EUR, respectively. Due to technical problems with the training tasks included in the first post-test, the data of three participants were not registered (memory task: one matched control; speed task: one trainee from each group).

### Apparatus and Software

Presentation 13.0 software was used for stimulus presentation and response recordings during training. The training notebooks had 14-inch LCD displays (with a resolution of 1280×800 pixels). During the post-tests Inquisit 2.0 software was used, except for the training tasks, for which Presentation 13.0 software was used. The PCs were equipped with 17 inch color screens (with a resolution of 1280×1024 pixels).

### General Training Procedure

There were two different training procedures: one aimed to enhance face memory and the other to enhance speed of face cognition. Participants completed their first training sessions in groups in the presence of an experimenter. Subsequently, they practiced at home using the same notebook computer as in the first session. Reward points were given to improve motivation. At the end of the training, the points were converted into a monetary reward. Trainees were instructed to keep the time of day, place, and light situation for the training as constant as possible. Compliance was monitored via weekly mailings of electronic data from each session.

### Training Face Memory

#### Stimuli

**Figure 1:**
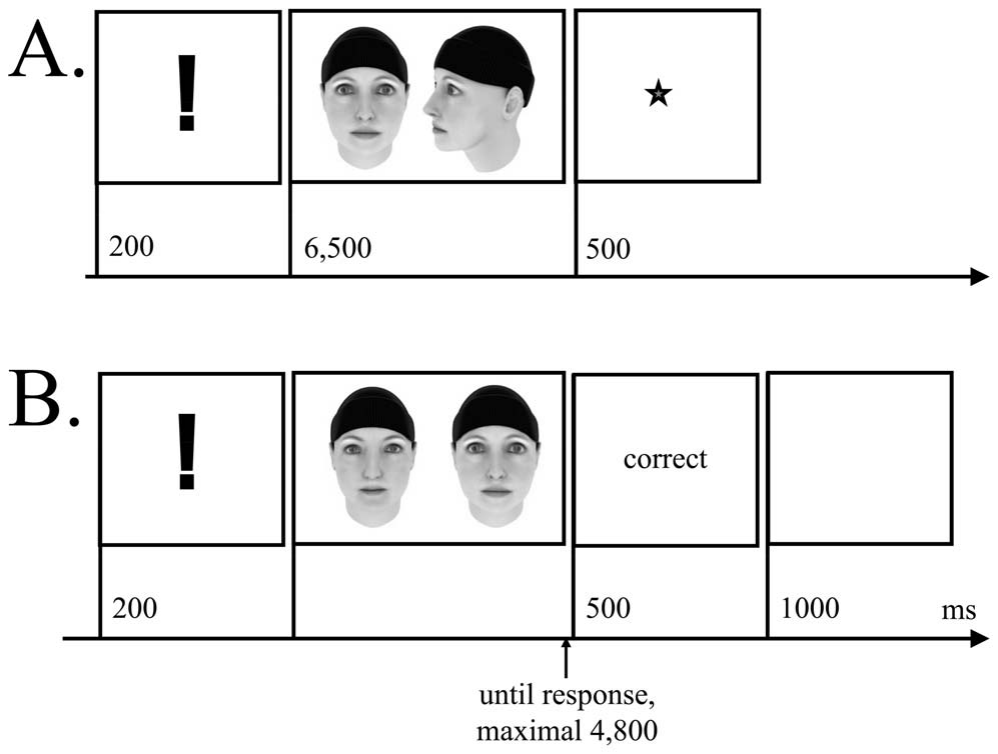
Trial sequences from the face memory training of a learn trial (Panel A), and of a test trial with feedback for a correct answer (Panel B).

**Figure 2:**
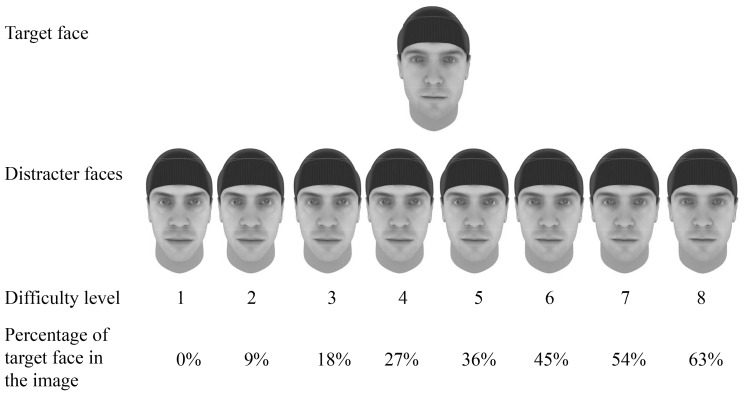
Examples of the face stimuli for the memory training task. Trainees memorized the target face in the top row. To create distracters for the subsequent test phase different amounts of the target were morphed into the images, ranging from Level 1 with 0% of target morphed into the image of the distracter to Level 8 with 63% of the target morphed into the distracter.

All stimuli for training were artificially generated faces (FaceGen Modeller 3.2). The parameter values for all generated faces were as follows: The age range was restricted to 20–40 years of age. The faces were generated randomly and checked individually before being saved because the program might produce extremely distinct faces. The variables are continuous. On the variable “Caricature” – ranging from “average” over “caricature” to “Monster” – only average to caricature level faces were accepted and all faces ranging between caricature to monster were rejected. On the variable “Asymmetry” – ranging from symmetric over typical to warped – symmetric and typical faces were accepted and warped faces were rejected. Female and male faces were equally represented and of neutral expression. None of the faces contained external features (hair, beards, earrings, or glasses) and all wore the same cap (cf. Figs. 1 and 2). For each training session, nine target faces were generated. For each target, four further faces were randomly produced in order to morph them with the target faces into distracters. Face models were imported into Cinema 4D 11.0 software. Each target model was morphed with 9 different amounts of the distracter faces. Morphs were created only from faces of the same sex and were rendered in three views: frontal, 30° and 60° left profiles. This produced a total of 972 images per session (9 targets ×4 faces for morphing of distracters ×9 morph combinations ×3 views).

All face images were shown on a white background and scaled to 400×400 pixels (82×82 mm). They were freely viewed at a distance of about 50 cm, subtending a visual angle of approximately 9.4°×9.4°.

#### Procedure

The training comprised a study block (Fig. 1A) followed by a filler task and six test blocks (Fig. 1B). The filler task was a general knowledge quiz, lasting 1.5 min. From the second session on, three blocks with faces learned in the previous session were administered before the study block.

#### *Task*

During each study block, 9 target faces had to be memorized. A study trial started with an exclamation mark presented for 200 ms, which was replaced by two images depicting the same face in frontal view and a 60° profile for 6.5 s. A star, shown for 500 ms, marked the end of the trial. The instruction encouraged to memorize both views as accurately as possible.

A test block comprised 18 trials presented in a two-alternative forced-choice paradigm with a familiarity task. Two trials for each target face were included. A test trial started with the presentation of an exclamation mark for 200 ms, followed by two faces, a target and a distracter face displayed until a response occurred or 4.8 s had passed. Trainees were asked to press a button on the keyboard on the side corresponding to the presentation of the target. The two faces were always of the same gender and depicted in the same view. Feedback was displayed for 500 ms using the German words for “correct” (richtig), “incorrect” (falsch), “faster, please” (schneller, bitte). For responses given within the first 200 ms after target presentation “do not guess, please” (bitte nicht raten) was displayed. The trial ended with a blank screen for 1 s (intertrial interval).

At the end of each block, feedback about performance in that block was given. The sum of hits, reward points scored in this block, and the level of difficulty for the next block were displayed. At the end of each session, participants were shown an overview of the training of that day. In the first and third block, recognition of the frontal view, in the second and fourth block recognition of the 60° views, and in the fifth and sixth block recognition of the 30° views was tested. The view of 30°, which had not be seen during learning, was included to ensure that faces and not only images had been learned [54].

#### *Adaptation*

A dynamic adaptation procedure aimed to maximize and to smooth the challenge across participants while keeping their motivation high. Different levels of difficulty were created by morphing different amounts of the target face with the distracter (see Fig. 2). At Level 1 (easiest), there was no morphing and thus a completely different face was used as distracter. For Levels 2 to 8, increasing proportions of the target face were combined with the distracter (Level 2 to 8: 9, 18, 27, 36, 45, 54, and 63% of target, respectively). Discrimination difficulty increased with the contribution of target face. The first test block was always at Level 3. The level for the following test blocks depended on the percentage of correct responses in the preceding block. Table 2 shows the adaptation steps of the difficulty levels. All adaptation steps remained within the range of Level 1 to Level 8. In the three test blocks with faces learned the previous day, the levels were not adapted but remained fixed to the levels of the previous day.

**Table 2:** Adaptation Steps for the Face Memory Training Task as a Function of the Percentage of Correct Responses.

Test block	Percentage correct in the preceding block	Difficulty level in this block
1st	for all	3
2nd	56% or less	1
	57–61%	2
	62–67%	3
	68–78%	4
	79–83%	5
	84–89%	6
	90–94%	7
	95–100%	8
3rd to 6th	67% or less	next lower
	68–83%	no change
	84% and more	next higher

Note. Adaptation started in the second test block.

#### *Reward points*

Two reward points were granted for each test block with 16 or more hits. At the end of each session, the sum of hits from the highest difficulty level was recorded as best achievement of this session and compared to the best achievements of the previous sessions. If it was the highest score so far, seven additional points were granted (with this modified score subsequently serving as new high score).

### Training Speed of Face Cognition

#### Stimuli

All stimuli were taken from the set created for the memory training task. For each session 45 faces were used with two images each (frontal view and 30° profile). Each face appeared up to five times within a session.

#### Procedure

**Figure 3:**
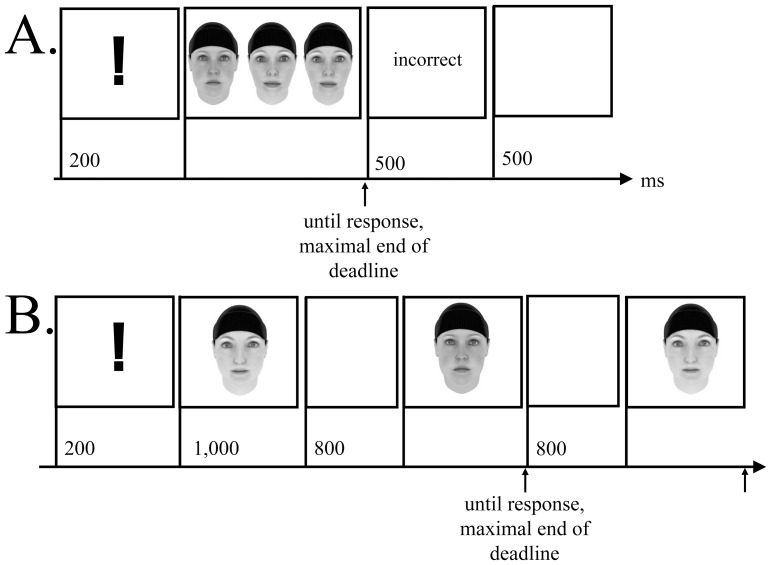
Trial sequences from the speed of face cognition training: odd-man-out task (Panel A) and 1-back task (Panel B).

Each training session consisted of two tasks, the odd-man-out (Fig. 3A) and the 1-back task (Fig. 3B), each with 12 blocks of 10 trials. Views of faces changed between blocks. At the beginning of each block, a deadline for reaction times (RT) was displayed. This deadline was adapted individually with a tracking algorithm (for details, see Table 3). Instructions emphasized accurate responses within the deadline. At the end of each task, participants were shown an overview of their performance.

**Table 3:** Adaptation Steps for the Deadline of the Speed of Face Cognition Tasks as a Function of the Percentage of Correct Responses.

Block	Percentage of correct responses	Adaptation steps of the response deadline	
		1st session	Following sessions
1st	60% or less	+400	+200
	61–70%	+200	+100
	71–80%	+100	+50
	81–90%	0	0
	91–100%	−200	−100
2nd–12th	55% or less	+240	+120
	56–65%	+180	+90
	66–75%	+120	+60
	76–85%	+60	+30
	86–95%	0	0
	96–100%	−60	−30

Note. The deadline for the first block of the first session was 2000 ms. In the following sessions, the deadline for the first block was calculated as 200% of the grand average, mean RT of the previous session, but with a maximum of 2000 ms.

#### *Odd-man-out task*

Each trial began with the presentation of an exclamation mark for 200 ms, followed by three faces presented side-by-side, shown until a response was given or the end of the deadline. Two of the faces were identical and the third face was the odd-man. The odd-man appeared either on the left or the right side of the screen. This position was randomized from trial to trial. Trainees responded by pressing one of two buttons on the keyboard on the side corresponding to the presentation of the odd-man. Only negative feedback was given using the German words for “incorrect” (falsch), “faster, please” (schneller, bitte), and for “do not guess, please” (bitte nicht raten). Feedback was displayed for 500 ms, thus these trials were of longer duration than correct trials. Every trial ended with a blank screen for 500 ms (intertrial interval).

#### *1-back task*

Blocks began with the presentation of a centered exclamation mark for 200 ms, followed by the first face, presented for 1000 ms, and then a blank screen for 800 ms. The following 10 faces were targets. Participants decided whether the current face was the same as the preceding one. They pressed a left or right button on the keyboard for same or different faces, respectively. Faces remained on display until the response was given or to the end of the deadline. The feedbacks were the same as in the odd-man-out task. Every trial ended with a blank screen for 800 ms (intertrial interval).

#### *Adaptation*

The first session started with a response deadline of 2,000 ms. Responses were considered correct only if the appropriate key was pressed within the deadline. In the first session, the deadline was adapted in large steps to bring everyone quickly to their individual achievement level. Table 3 presents the steps used to adapt the deadline in both training tasks for face cognition speed. The adaptation steps were large after the first block. In the following blocks, the steps depended on the percentage of correct responses in the two preceding blocks.

#### *Reward points*

Two reward points were granted for 90% or more correct responses within the deadline in the preceding two blocks. At the end of each session, the mean RT for each task was recorded and compared to the mean RTs of the previous sessions. If it was the fastest mean RT for this task so far, an additional five points were granted.

### Post-Tests

Both post-tests were abridged, three-hour versions of the test battery administered as pre-test. These tests were conducted as small group sessions at the Department of Psychology of the Humboldt-University at Berlin. Detailed information about the pre-test can be found in Hildebrandt et al. [55]. A brief description of all tasks included in the post-tests is provided in the (File S1). The post-tests included 14 face cognition tasks, 4 indicators of object cognition, a questionnaire on face cognition skills, and one indicator each for general cognition (*General cognitive ability* [56]), immediate and delayed memory (*Immediate memory* and *Delayed memory* – verbal memory IDM3–IDM4 from the Wechsler Memory Scale [57]), and mental speed (*Finding As* [58]). At the end of the post-test session, the memory training task and the odd man out task from the speed training were administered to measure task specific training effects. To render the outcome measures better comparable to the test battery, the training tasks were not adaptive in the post-tests: The memory task was administered at difficulty level three. From the speed task, the reaction deadline was removed.

Participants from the two intervention groups had finished their training on average 2.8 days before the first post-test (range: 0 to 9 days). This interval did not differ between the training groups, *F*<1.7. The second post-test was administered on average 94.5 days after the end of the training (range: 75 to 99 days). This interval did not differ between the two groups, *F*<1. The second post-test consisted of the same set of tasks as the first post-test and was conducted with the same apparatus.

### Data Preparation and Analysis

#### Manifest level

Only correct responses given at least 201 ms after target onset were analyzed. Performance was scored as proportion of correct responses for all face perception tasks, all face memory tasks, as well as for indicators of object perception, immediate and delayed memory, and general ability. Performance was scored as RTs for the speed of face cognition tasks, indicators of object cognition speed, and the indicator of mental speed. RTs were winsorized (e.g., [59]) as follows. For trials 3.5 *SD*s slower than the individual mean, the latencies were trimmed by a recursive procedure that replaced these outliers with the mean value plus 3.5 *SD*s until there were no values above the mean plus 3.5 *SD*s (for the rationale of this data manipulation see Wilhelm et al. [5]. The trimmed RTs were transformed into inverted latencies by the formula 1000/RTs (in ms) in order to obtain a measure of correctly processed trials per second.

Data were analyzed to determine group differences, changes over time, and interactions. The change of performance during training was assessed with regression analyses. The training tasks included in the post-tests were analyzed with the between-subjects factor group (memory, speed, matched controls). Post-hoc comparisons were Bonferroni corrected (*N* = 2). For repeated measures Huynh-Feldt corrected analyses of variance [60] were performed and uncorrected degrees of freedom and corrected *p*-values are reported. For all other tasks, net effect sizes assessed change over time to control for practice effects due to retest. First, effect sizes were calculated for the three matched groups separately as mean pre-post differences of the indicators divided by the standard deviation at pre-test [41]. Next, net effects were calculated as the difference in effect size between each training group and the control group. The interaction of occasion (pre- vs. post-test) with group (each training group separately vs. control group) served as indicator of statistical significance.

#### Latent factor level

The effects of training were investigated at the ability level with structural equation models (SEM). SEM is a statistical technique for testing and estimating causal relationships in observed data. SEM encourages confirmatory rather than exploratory modeling. With an accepted theory or otherwise confirmed model—as was the case here, in which we started from established models of face cognition—SEM is used to estimate the values of free parameters by specifying one or several competing theoretical models a priori. Latent factors can represent abilities that are not measured directly but are estimated in the model from observed variables on the basis of the theoretical assumptions about which indicator (e.g., the performance in the indicator, acquisition curve) contributes to a particular underlying ability (e.g., face memory). Pairs of indicators, supposedly assessing the same ability (e.g., face memory), should— other things being equal— correlate higher with each other than with two indicators assessing different abilities (e.g., face memory and face perception). SEM allows for capturing the unreliability of measures in the model and accurately estimating the structural relations between latent factors. Given these methodological features and the research questions derived previously, SEM is the methodological tool of choice in the present context. Applying SEM, the estimated theoretical covariance matrices representing the relationships between variables in the model can be compared with the actual empirical covariance matrices [61], [62]. Various formal statistical tests and fit indices have been developed for this purpose. Because different measures capture different aspects of the model fit, it is appropriate to report several fit measures. Some of the more commonly used fit measures are the chi-square test, root-mean-square-error of approximation (RMSEA), and comparative fit index (CFI). A chi-square test is a fundamental measure of fit used in the social sciences.

Face cognition was modeled according to the three-factor model suggested by Wilhelm et al. [5]. To analyze training effects for each ability of face cognition separately, autoregressive change models were calculated [63]. The significance of correlations was evaluated by the critical ratio (*C.R.*). An estimate is significant at the .05 level if the critical ratio exceeds the value of 1.96 [64]. Changes at the latent level may be analyzed by comparing the means over time only if measurement invariance has been established. For models not invariant over time, changes were analyzed by regressing dummy variables for the respective training group onto the latent factor [65]. All analyses at the latent level were computed with Mplus 5 [66]. The influence of the training on the latent variable was evaluated by fixing the effect to zero and comparing the model fit with the chi-square difference test. The differences in chi-squares (Δχ2) and in their degrees of freedom (Δdf) test the null hypothesis that the restricted model fits the data as well as the less restrictive model [64]. If there is a significant loss of fit due to the restriction, it suggests an influence of the training.

Model fit was evaluated using the chi-square test, the comparative fit index (CFI), the root-mean-square error of approximation (RMSEA), and the standardized root-mean-square residual (SRMR). In the SEM approach the chi-square test is a function of the sample size and the difference between the observed covariance matrix and the theoretical model covariance matrix. The significance level of the chi-square test is compared with the corresponding value of the chi-square distribution. Competing models are frequently compared by evaluating their chi-square values and their degrees of freedom. The CFI is derived from a comparison of a hypothesized model with the independence model taking the sample size into account; values of .95 or larger indicate excellent fit, whereas CFI values below .90 are frequently deemed unacceptable and therefore lead to the rejection of the model. The RMSEA accounts for the error of approximation in the population and is sensitive to model complexity. Values less than .05 indicate good fit, and values up to .08 represent reasonable approximation errors in the population, whereas values higher than .08 are usually considered as indicating unacceptable fit. However, if sample size is small, RMSEA tends to reject true-population models [67]. SRMR is the standardized difference between the observed covariance and the predicted covariance; a value of less than .08 is considered to indicate good fit.

#### Testing model invariance

Training is expected to influence the factor scores indicating intrinsic or quantitative within-person changes [63]. The quality of model fit in confirmatory factor analysis can also be expressed by comparing competing models via likelihood ratio tests (e.g., by constraining correlations or factor loadings). If the introduction of constraints (e.g., the correlation between latent factors for memory and perception is fixed to be one) causes a significant decline of the model fit, one should consider accepting the less parsimonious model (e.g., a model in which perception and memory indicators load on two not perfectly correlated factors). The comparison of the model fits can be based on a chi-square distributed test value by taking into account the difference between the chi-square values and the difference of the degrees of freedom in the competing models.

First, the invariance of factor loadings (configural invariance) over time is tested because the intervention procedure itself could have altered the basic meaning of the common factors [68]. Second, metric or weak invariance constrains factor loadings to equality and implies equal regression slopes over time. Metric invariance is achieved if the strengths of the relation between specific scale items and the underlying constructs do not differ over time [69]. Third, scale or strong invariance is investigated by additionally constraining the intercepts of the factor loadings to equality [69]. Nested models were compared with the chi-square difference test. If no significant loss of fit was established, it was taken as support of the assumption of equality.

## Results

For reasons of comparison, the effect sizes are all reported as Cohen's *f*. Effect sizes of .10, .25, and .40 are considered as small, medium, and large effect sizes, respectively [70].

### Performance during Training

**Figure 4:**
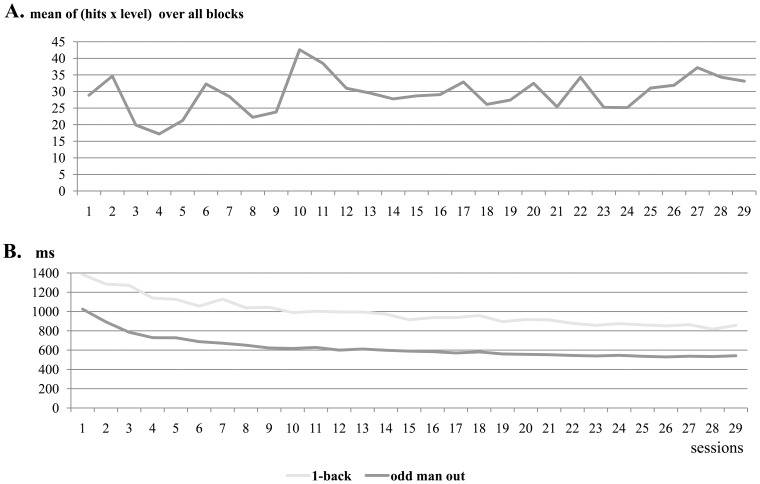
Trainees' performance over the courses of training for face memory (Panel A) and for facial speed (Panel B).

Each training procedure consisted of 29 sessions (Fig. 4). Fifteen of the memory trainees completed all sessions and the other 4 trainees completed 28 sessions. Eighteen speed trainees completed all sessions, one completed 28 sessions, and one 27 sessions.

To capture the influence of the changing difficulty levels on the achieved hits over the course of the memory training, we report the product of hits and level of difficulty (i.e., morphing level). A marginally significant standardized regression coefficient of *b* = .362, *t*(28) = 2.05, *p* = .05, *f* = .388, indicated that there was an overall trend for an increase in performance during the memory training. The significant standardized regression coefficients in the analyses of the speed training for the odd-man-out of *b* = −.912, *t*(27) = −11.55, *p*<.001, *f* = 2.225, and for the 1-back task of *b* = −.832, *t*(27) = −7.78, *p*<.001, *f* = 1.495, indicated that RTs declined during training.

### First Post-Test

Table 4 shows means and standard deviations for all tasks administered in the first post-test for both practice groups and both control groups.

**Table 4:** Means and Standard Deviations of Behavioral Data for Practice and Control Groups in the Pre-Test and the First Post-Test.

	Practice groups				Control groups			
	Memory		Speed		Matched		Unmatched	
	Pre-test	Post-test	Pre-test	Post-test	Pre-test	Post-test	Pre-test	Post-test
Trained memory task (TRM)	-	.57 (.10)	-	.53 (.06)	-	.62 (.08)	-	.62 (.11)
Trained speed task, odd-man-out (TRS)	-	.75 (.14)	-	1.09 (.21)	-	.76 (.20)	-	.70 (.17)
Facial resemblance (FP1)	.68 (.08)	.74 (.06)	.70 (.10)	.69 (.09)	.73 (.10)	.75 (.08)	.70 (.09)	.74 (.09)
Sequential matching of part-whole faces – condition part (FP2)	.74 (.08)	.75 (.09)	.72 (.08)	.68 (.10)	.72 (.08)	.74 (.06)	.70 (.09)	.68 (.13)
Sequential matching of part-whole faces – condition whole (FP3)	.70 (.11)	.71 (.10)	.68 (.10)	.67 (.11)	.68 (.07)	.72 (.13)	.68 (.13)	.67 (.12)
Simultaneous matching of spatially manipulated faces – condition upright (FP4)	.75 (.15)	.75 (.12)	.75 (.14)	.73 (.12)	.78 (.09)	.74 (.12)	.75 (.12)	.73 (.13)
Simultaneous matching of spatially manipulated faces – condition inverted (FP5)	.66 (.11)	.68 (.11)	.66 (.12)	.61 (.10)	.64 (.10)	.66 (.13)	.64 (.11)	.63 (.12)
Acquisition curve (FM1)	.91 (.06)	.93 (.07)	.90 (.07)	.90 (.08)	.90 (.09)	.94 (.06)	.88 (.08)	.91 (.08)
Decay rate of learned faces (FM2)	.88 (.07)	.89 (.08)	.87 (.09)	.86 (.12)	.86 (.10)	.91 (.07)	.85 (.12)	.88 (.12)
Eyewitness testimony (FM3)	.74 (.09)	.76 (.09)	.71 (.09)	.73 (.10)	.77 (.07)	.76 (.12)	.73 (.11)	.75 (.11)
Recognition speed of learned faces (SFC1)	.78 (.14)	.86 (.16)	.79 (.15)	1.06 (.21)	.81 (.20)	.86 (.24)	.77 (.18)	.81 (.22)
Delayed non-matching to sample (SFC2)	.98 (.20)	.97 (.17)	.99 (.23)	1.09 (.18)	.97 (.24)	.91 (.19)	.95 (.24)	.90 (.22)
Simultaneous matching offaces from different viewpoints (SFC3)	.56 (.13)	.63 (.15)	.57 (.15)	.78 (.19)	.54 (.16)	.57 (.13)	.56 (.19)	.57 (.21)
Simultaneous matching of upper face-halves – condition aligned (SFC4)	.68 (.17)	.69 (.14)	.65 (.14)	.91 (.12)	.65 (.22)	.70 (.23)	.62 (.20)	.67 (.22)
Simultaneous matching of upper face-halves – condition non-aligned (SFC5)	.70 (.17)	.70 (.13)	.67 (.14)	.92 (.13)	.65 (.21)	.71 (.22)	.63 (.21)	.68 (23)
Simultaneous matching of morphs (SFC6)	.68 (.14)	.72 (.12)	.69 (.12)	.84 (.17)	.70 (.20)	.72 (.20)	.66 (.19)	.66 (20)
Sequential matching of part-whole houses – condition part (OC1)	.77 (.08)	.78 (.11)	.74 (.09)	.72 (.10)	.73 (.12)	.73 (.08)	.70 (.11)	.73 (.10)
Sequential matching of part-whole houses – condition whole (OC2)	.71 (.08)	.72 (.09)	.68 (.10)	.63 (.11)	.67 (.11)	.71 (.13)	.67 (.11)	.68 (.11)
Delayed non-matching to sample of houses (OC3)	.90 (.19)	.90 (.15)	.85 (.17)	1.01 (.20)	.82 (.20)	.83 (.16)	.81 (.19)	.83 (.19)
Simultaneous matching of houses (OC4)	.67 (.16)	.62 (.12)	.61 (.17)	.73 (.18)	.64 (.20)	.70 (.33)	.63 (.21)	.60 (.20)
Immediate memory (GA1)	.78 (.12)	.80 (.17)	.74 (.14)	.74 (.21)	.72 (.16)	.79 (.15)	.77 (.12)	.80 (.14)
Delayed memory (GA2)	.89 (.14)	.91 (.14)	.85 (.18)	.89 (.17)	.82 (.19)	.89 (.13)	.88 (.11)	.87 (.19)
General cognitive ability (GA3)	.42 (.19)	.41 (.19)	.36 (.18)	.31 (.21)	.43 (.18)	.38 (.19)	.46 (.17)	.37 (.21)
Mental speed (GA4)	1.65 (.27)	1.65 (.21)	1.66 (.30)	1.84 (.25)	1.65 (.28)	1.63 (.29)	1.60 (.26)	1.56 (.25)

Note. Estimated values for accuracy tasks (TRM, FP1-5, FM1-3, OC1-2, GA1-3) are mean accuracies and for speed tasks (TRS, SFC1-6, OC3-4, GA4) inverted RTs, calculated as 1000/RT in ms; *SD*s are shown in parentheses.

#### Trained Tasks

The trained memory task was difficult as indicated by the low performance in all groups. Performance was above guessing rate of 50% for the memory and the control group, *t*(17) = 2.81, *p*<.05, and *t*(18) = 6.20, *p*<.001, respectively, whereas the speed group performed at chance, *t*(18) = 2.10, *p* = .051, *d* = .5. ANOVA revealed a main effect of group, *F*(3, 111) = 5.15, *p*<.01, *f* = .373; pairwise comparisons indicated that the memory group did not differ from the other two groups, *p*s>.29, whereas the speed group performed significantly less accurate in comparison to the matched control group, *F*(1, 53) = 5.06, *p*<.01, *f* = .309.

On the trained speed task, all participants performed with high accuracy as required for a speed task. Mean RTs were 1550 ms (*SD* = 358 ms) for the memory group, 1034 ms (*SD* = 220 ms) for the speed group, and 1622 ms (*SD* = 487 ms) for the matched control group. The RTs of the three groups differed significantly, *F*(2, 53) = 14.04, *p*<0.001, *f* = .728. Pairwise comparisons revealed that the speed group showed shorter RTs than both the memory group, *F*(1, 35) = 28.15, *p*<.001, *f* = .867, and the control group, *F*(1, 37) = 23.18, *p*<.001, *f* = .792. The memory and control groups did not differ, *F*<1.

To find out if any pre-test variables predicted the speed training gains, we analyzed the following variables: age, factor scores in the pre-test on face perception, on face memory, on speed of face cognition, on general cognition, on mental speed, and on immediate and delayed memory. None of the regression analyses with these variables as predictors were significant, *p*s>.18. The variable gender almost reached significance, *b* = −.472, *t*(19) = −2.13, *p* = .054, indicating that women tended to improve more than men. With the caution appropriate for the small *N*, we take our results to hint that performance improvement for speed of face cognition is not restricted to a certain population.

Both trained tasks fulfilled the criteria for reliable measures as indicated by high internal consistencies, Cronbach's αs were .812 for the memory task and .918 for the speed task. Performance on the speed training task in the first post-test correlated highly with the indicators of speed of face cognition from the test battery (all tasks *r*s>.69, *p*s<.01), confirming that the speed training task fitted well with the other indicator tasks of speed of face cognition. The correlations of the memory training task with the indicators of face memory from the test battery were rather low (*r*s<.31), indicating that it might not have measured the same ability or that it was much more difficult than the other indicators.

In sum, in the first post-test the memory group did not perform better than the other groups on the trained face memory task. In contrast, the speed group responded significantly faster on the trained speed task than the other groups.

#### Manifest Level Analysis

**Figure 5:**
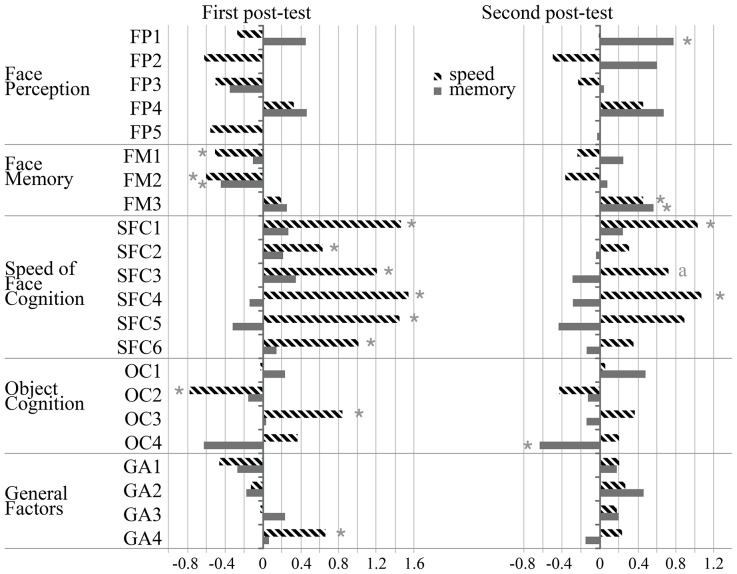
Performance gains from pre-test to first and second post-test as net effect sizes. Bars depict net effect sizes (difference in standardized changes between the experimental and the control group), for the group trained in face memory (solid bars) and in speed of face cognition (pattern filled bars). Statistical significance was tested as interactions (* *p*<.05; a: *p* = .052) between group (training vs. control) and occasion (pre- vs. post-test). FP1 – Facial resemblance; FP2 – Sequential matching of part-whole faces – condition part; FP3 – Sequential matching of part-whole faces – condition whole; FP4 – Simultaneous matching of spatially manipulated faces – condition upright; FP5 – Simultaneous matching of spatially manipulated faces – condition inverted; FM1 – Acquisition curve; FM2 – Decay rate of learned faces 1; FM3 – Eyewitness testimony; SFC1 – Recognition speed of learned faces; SFC2 – Delayed non-matching to sample; SFC3 – Simultaneous matching of faces from different viewpoints; SFC4 – Simultaneous matching of upper face-halves – condition aligned; SFC5 – Simultaneous matching of upper face-halves – condition non-aligned; SFC6 – Simultaneous matching of morphs; OC1 – Sequential matching of part-whole houses – condition part; OC2 – Sequential matching of part-whole houses – condition whole; OC3 – Delayed non-matching of houses to sample; OC4 – Simultaneous matching of house morphs; GA1 – Immediate memory; GA2 – Delayed memory; GA3 – General cognitive ability; GA4 – Mental speed.

For each indicator, training effects were calculated and significance was tested as the interaction of occasion (pre- vs. post-test) with group (each training group separately vs. control group). Figure 5 depicts effect sizes for both post-tests.

#### *Face Tasks*

Performance results for all tasks are summarized in Table 4. Mean performance was clearly above chance of 50% for all tasks. Figure 5 depicts net effect sizes for the tasks of the first post-test (left column). There were no significant positive net effects for the memory-trained group, *F*s<2.2, and one negative net effect size for the FM2 indicator of face memory, *F*(1, 37) = 6.45, *p*<.05. The speed-trained group performed significantly better on all indicator tasks for speed of face cognition, all *F*s(1, 38)>7.9, *p*s<.01, *f*s>.457. The speed group showed significant negative effect sizes for two face memory tasks, for FM1, *F*(1, 38) = 7.25, *p*<.05, and for FM2, *F*(1, 38) = 5.87, *p*<.05.

In sum, the results indicated reaction time reductions from pre- to post-test for the speed trained group. There was no increase in performance for the memory-trained group.

#### *Object Tasks*

There were no significant net effects on the house tasks for the memory trained group, *F*s<1.97. For both tasks measuring object cognition speed, the net effect sizes for the speed-trained group were positive, but were significant only for the indicator OC3, *F*(1, 38) = 7.46, *p* = .01, *f* = .443. Further, there was a significant negative effect size for the object perception task OC2 in the speed group, *F*(1, 38) = 4.96, *p*<.05, *f* = .361.

#### *Further Indicators*

There were no significant net effect sizes for the indicator tasks of immediate and delayed memory or general cognitive ability, *F*s<1.2. The speed-trained group achieved a significant positive effect size on the task measuring mental speed, *F*(1, 38) = 6.22, *p*<.05, *f* = .405, indicating far transfer from the speed training task to the general mental speed ability.

Taken together, on the manifest level, only the training of facial speed was effective. It did not transfer to other indicators of face cognition but enhanced performance on all other indicator tasks of speed, that is, on tasks for object speed and for mental speed.

#### Latent Factor Analysis

The results of data modeling are based on results of all four groups with a total of 118 participants. Generally, error terms were uncorrelated. Some indicators, however, comprised different experimental conditions of the same task (*Sequential matching of part-whole faces: part and whole, Simultaneous matching of spatially manipulated faces: upright and inverted*, and *Simultaneous matching of upper face-halves: aligned and non-aligned*). For these indicators correlations of error terms were theoretically expected and therefore specified in the models. The following sections apply the established measurement model with three ability factors of face cognition to the post-test data. To test invariance over time, nested models were specified for each of the three abilities. Next, an omnibus model including all factors and measurement occasions was computed. Finally, the specificity of the effects of the speed of face cognition training was tested further with an extended post-test model.

#### Measurement Model of the Post-Test

The measurement model of the post-test including all three ability factors of face cognition had an acceptable fit, *χ* ^2^(96, N = 118) = 158.36, CFI = .950, RMSEA = .074, SRMR = .063. All factor loadings were moderate (.38 to .94) as were the correlations of the abilities (.37 to .78). The non-significant Δ*χ* ^2^-test of .1 corresponding to Δ*df* = 1 [64] indicated the regression of the group that trained memory onto the memory factor was not significant. Contrarily, the regression of the group that trained speed onto the speed factor was significant, as confirmed by a significant Δ*χ* ^2^-test of 25 corresponding to Δ*df* = 1, indicating that speed training influenced the targeted ability. All other regressions of group onto the abilities at post-test were not significant, Δ*χ* ^2^(1)<3.

#### Testing Invariance over Time

A series of models with sequentially added restrictions was specified for each ability factor. Table 5 summarizes the fit indices for the nested models testing invariance over time.

**Table 5:** Competing Structural Equation Models Investigating Training-Induced Changes of Face Perception, Face Memory, and Speed of Face Cognition at the Latent Factor Level.

Model	Specifications	*χ* ^2^	df	*p*	CFI	RMSEA	SRMR
Perception							
**P1**	**2 factors, indicators correlated over time**	**50.81**	**26**	**.003**	**.927**	**.090**	**.121**
P2	2 factors, all factor loadings constrained	66.24	31	.000	.897	.098	.181
P3	2 factors, loadings and intercepts constrained	83.11	36	.000	.862	.105	.174
Memory							
**M1**	**2 factors, indicators correlated over time**	**12,81**	**10**	**.234**	**.995**	**.049**	**.038**
M2	2 factors, all factor loadings constrained	77,82	13	.000	.886	.206	.583
M3	2 factors, loadings and intercepts constrained	90,42	16	.000	.869	.199	.560
Speed							
**S1**	**2 factors, indicators correlated over time**	**88.66**	**56**	**.004**	**.981**	**.070**	**.035**
S2	2 factors, all factor loadings constrained	142.04	62	.000	.953	.105	.185
S3	2 factors, loadings and intercepts constrained	184.95	68	.000	.931	.121	.180

Note. CFI = comparative fit index; RMSEA = root-mean-square error of approximation; SRMR = standardized root-mean-square residual; bold demarcates the final models.

#### Testing Invariance over Time for Face Perception

As a first step, the post-test factor of perception was regressed onto the pre-test factor and the tasks were autocorrelated over time (Model P1). This model had an almost acceptable fit. Constraining the factor loadings to equality (Model P2) reduced the model fit, as confirmed by a significant test of Δ*χ* ^2^(5) = 15, and had to be rejected. As did further constraining the intercepts to equality over time (Model P3), Δ*χ* ^2^(5) = 17.

For face perception the same number of factors could be established over time but not the same pattern of loadings. Metric invariance was rejected, and Model P1 was the final model. The standardized factor loadings were substantial (.42 to .71). The autocorrelations of the unique scores over time were significant for three indicators but rather low (.20, .24, and .25) and not significant for two other indicators. Also, two indicators consisted of two conditions and these conditions were expected to correlate within the test session but did not. Overall, this model does not fit the data well. The regressions of the memory and the speed training group onto the post-test factor of the final model were not significant, Δ*χ* ^2^(1) = 2, indicating that, as expected, neither of the training procedures influenced face perception.

#### Testing Invariance over Time for Face Memory

The baseline model with tasks correlated over time (Model M1) had an acceptable fit. Constraining the factor loadings to equality (Model M2) decreased the model fit significantly (Δ*χ* ^2^(3) = 65). Further, constraining the intercepts of the indicators to equality (Model M3) led to an unacceptable model fit (Δ*χ* ^2^(3) = 13). In a model with unequal loadings, the difference in the latent means of the two time points might be confounded with differences in the regression slopes over time. For such cases Byrne et al. [71] proposed comparing latent means under partial invariance. This procedure assumes that the non-invariant item will not affect the latent means comparison to a great extent. But it is important to keep in mind that such results are explorative and might reflect an attribute of the sample rather than describe the theoretical model. There were only three indicator tasks for the latent memory factor. We therefore refrained from proceeding in the suggested explorative fashion, since this would change the model strongly. Metric invariance was rejected.

Model M1 was the final model. All standardized factor loadings were substantial (.62 to .95). The autocorrelation of the unique scores over time was significant only for the Eyewitness testimony task but not for the tasks *Acquisition curve* or *Decay rate of learned faces*. The regression of the memory training group onto the post-test factor for face memory was not significant, Δχ2(1) = 1, indicating that memory training did not influence the latent ability of face memory. However, the regression of the speed training group was significant and negative, Δχ2(1) = 11, indicating that the speed training had a negative influence on the memory scores at post-test.

#### Testing invariance over time for speed of face cognition

For the speed of face cognition factor, the baseline model (Model S1) with tasks correlated over time fit the data well. Constraining the factor loadings to equality (Model S2) however had to be rejected, as implied by a significant Δ*χ* ^2^-test of 53, Δ*df* = 6. In Model S3, the intercepts of the indicator tasks were constrained to equality, but this resulted in further loss of fit. Six tasks served as indicators for the facial speed factor. Testing partial invariance [71] revealed that even if the three tasks with the largest differences in factor loadings between pre- and post-test were excluded from the equality constraint and allowed to be estimated freely, there was still a significant loss of model fit, Δ*χ* ^2^(3) = 40. Therefore, this explorative method was abandoned and the strictly confirmatory Model S1 was accepted as the final model.

In model S1, all standardized factor loadings were substantial (.71 to .88). The autocorrelations of the unique scores over time were significant for three of six tasks. For the *Simultaneous matching of faces from different viewpoints* task the parameter almost reached significance, *C.R.* = 1.88, *p* = .060. The tasks *Simultaneous matching of upper face-halves – aligned* and *non-aligned* are two conditions of one assignment. As theoretically expected these two conditions were significantly correlated within each test occasion, *C.R*.s = 70.82 and 32.23 for pre- and post-test, respectively. The regression of the speed training group onto the post-test factor for speed of face cognition was significant, as indicated by a loss of fit when constraining this path to zero, Δ*χ* ^2^(1) = 53, indicating that the speed of face cognition training group scored higher on the post-test than the other groups. Regression of the memory training group was not significant, Δ*χ* ^2^(1) = 0, indicating that the memory training did not influence the latent factor for speed of face cognition.

We further tested if the changes in factor loadings between the two occasions were caused by the speed training. For this purpose, the final pre-post-model of speed of face cognition was calculated without the speed trainees. The model fit was reasonable, *χ* ^2^(45, N = 98) = 72.37, CFI = .981, RMSEA = .079, SRMR = .033. Exclusion of participants trained on speed still led to the rejection of equal factor loadings, Δ*χ* ^2^(6) = 43. Taken together these results indicate that the differences in factor loadings were not solely caused by the speed training.

#### Omnibus Model

**Figure 6:**
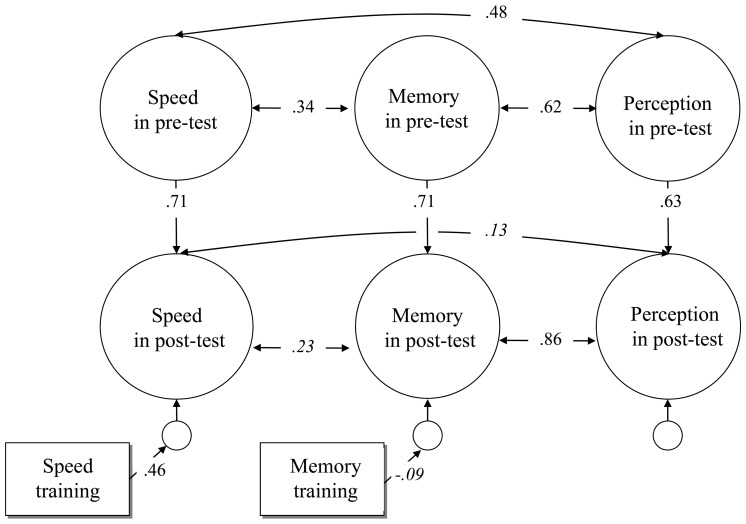
Omnibus model comprising pre- and post-test measurements for all three ability factors of face cognition with both training groups included as dummy variables. Unique scores were autocorrelated over time. Coefficients that did not reach statistical significance at α = .05 are italicized.

The omnibus model (Fig. 6) was composed of all three ability factors at pre- and at post-test. The model fit was assessed according to Hair et al. [72]. This model had an acceptable fit, χ^2^(382, N = 118) = 583.34, CFI = .928, RMSEA = .067, SRMR = .172. The factor loadings were correlated over time. The loadings of the pre- and post-factors for face memory and speed of face cognition showed considerable communalities (.54 to .91), whereas the loadings of the perception factors were weaker (.44 to .71). The correlations of the latent ability factors at pre-test and the correlation of perception with memory at post-test were substantial (.34 to .62). The latent speed factor at post-test did not correlate with the other factors. The regression of the memory group onto the latent ability of face memory was not significant, Δ*χ* ^2^(1) = 2. In contrast, the regression of the speed group onto the latent factor of speed of face cognition was meaningful, Δ*χ* ^2^(1) = 18, confirming that the speed training influenced the targeted ability. Regression of the speed group onto the memory factor at post-test was significant, Δ*χ* ^2^(1) = 12. All other regressions of group onto the abilities at post-test were not significant, Δ*χ* ^2^(1)< = 2.

#### Testing specificity of speed of face cognition training effects

To assess the influence of training the speed of face cognition on the speed of object cognition, the three-factor model of the post-test was extended. A new latent factor for speed of object cognition, composed of the two object speed tasks *Delayed non-matching of houses to sample* and *Simultaneous matching of house morphs*, was added to the model. The new model was estimated with four correlated latent factors, namely face perception, face memory, speed of face cognition, and speed of object cognition. The model fit the data well, *χ* ^2^(95, N = 118) = 156.26, CFI = .955, RMSEA = .074, SRMR = .057. All factor loadings were substantial (.38 to .95). The correlation of the two speed factors for faces and for objects was high (*r* = .97), thus, strongly indicating that the two factors might capture the same domain-general ability. The regressions of the speed group onto the two speed factors were significant, Δ*χ* ^2^(1) = 20 for speed of face cognition and Δ*χ* ^2^(1) = 14 for object speed, indicating that the speed training influenced both abilities. All other regressions of group onto the ability factors were not significant, Δ*χ* ^2^(1)<3.

To test the assumption of a domain-general speed ability, a competing model was estimated. In this model, the two latent speed factors were merged into one. The fit of the model with the domain-general latent speed factor was comparable to the model with two speed factors, *χ* ^2^(98, N = 118) = 159.21, *df* = 98, *p*<.000, CFI = .955, RMSEA = .073, SRMR = .057. The non-significant Δ*χ* ^2^-test of 2.95, Δ*df* = 3, indicated that no differentiation between speed of face and of object cognition was needed. Post-hoc the same models were calculated for the pre-test data. Their comparison also substantiated the irrelevance of the distinction between face and object speed.

#### Summary of the latent factor analyses

At the latent level, there was no increase of performance due to the memory training. In contrast, all models confirmed shorter RTs for the speed trainees. The speed of face cognition training also enhanced performance on a latent factor for object speed.

### Second Post-Test

The second post-test was conducted to assess the persistence of training effects over time at the manifest level. Only the three matched groups participated in this test. Because at pre-test the training tasks had not been administered, change of performance between the first and the second post-test was analyzed with repeated measures on the factor test occasion (first post-test, second post-test) and the between-subjects factor group (memory, speed, matched controls). For all other tasks, net effect sizes assessed change over time and were calculated in the same manner as for the first post-test. Statistical significance was tested with the interaction of occasion (pre-test vs. second post-test) and group (each training group separately vs. control group). For brevity, only results concerning the speed training will be reported because as indicated by the results of the first post-test this was the only effective training procedure. Table 6 summarizes the means and standard deviations for all speed tasks administered in the second post-test.

**Table 6:** Means and Standard Deviations of Behavioral Data from all Speed Tasks for the Three Matched Groups in the Second Post-Test.

	Practice groups		Control group
	Memory	Speed	Matched
Trained speed task, odd-man-out (TRS)	.75 (.15)	.96 (.19)	.84 (.35)
Recognition speed of learned faces (SFC1)	.91 (.16)	1.04 (.22)	.94 (.18)
Delayed non-matching to sample (SFC2)	.99 (.16)	1.09 (.25)	.99 (.27)
Simultaneous matching of faces from different viewpoints (SFC3)	.61 (.14)	.79 (.22)	.65 (.20)
Simultaneous matching of upper face-halves – condition aligned (SFC4)	.74 (.15)	.89 (.17)	.79 (.22)
Simultaneous matching of upper face-halves – condition non-aligned (SFC5)	.75 (.15)	.90 (.17)	.80 (.20)
Simultaneous matching of morphs (SFC6)	.72 (.13)	.79 (.20)	.78 (.26)
Delayed non-matching of houses to sample (OC3)	.93 (.19)	.98 (.20)	.90 (.19)
Simultaneous matching of house morphs (OC4)	.61 (.15)	.69 (.23)	.69 (.27)
Mental speed (GA4)	1.67 (.24)	1.80 (.25)	1.72 (.26)

Note. Estimated values are mean inverted RTs, calculated as 1000/RT in ms; SDs are shown in parentheses.

#### Trained speed task

For the speed training task, mean RTs did not differ between the two test occasions, *F*<1. However, there was a main effect of group, *F*(2, 54) = 9.82, *p*<.001, *f* = .603, and an interaction of group with test occasion, *F*(2, 54) = 8.78, *p*<.001, *f* = .570. Pairwise comparisons of the groups revealed that in the first post-test the speed group responded faster than the other two groups, *F*(1, 35) = 33.95, *p*<.001, *f* = .984, compared with the memory group and *F*(1, 37) = 24.35, *p*<.001, *f* = .810, with the matched controls. In the second post-test, only the difference to the memory group was still significant, *F*(1, 35) = 15.03, *p*<.001, *f* = .655. RTs of the memory group and the matched controls differed neither in the first nor in the second post-test. Comparing the two test occasions, the control group reacted faster in the second than in the first post-test, *F*(1, 54) = 6.11, *p*<.05, *f* = .336, whereas the speed group reacted significantly slower on the second post-test, *F*(1, 54) = 11.68, *p*<.001, *f* = .465. All other post-hoc comparisons were not significant, *F*s<1.9.

In the second post-test, correlations between the speed training task and the indicators of speed of face cognition from the test battery were strong (all tasks *r*s>.54, *p*s<.01), though weaker than in the first post-test.

#### Other speed tasks

Although for all speed of face cognition tasks the net effect sizes for the speed group were positive, only two of them were significant, *F*s(1, 38) = 8.84 and 6.38, *p*s<.05, *f*s = .482 and .410, for *Recognition speed of learned faces* and *Simultaneous matching of upper face-halves – condition aligned*, respectively. A third task, *Simultaneous matching of faces from different viewpoints*, displayed a trend, *F*(1, 38) = 4.04, *p* = .052, *f* = .326.

The memory group had a significant negative net effect size on the indicator of object speed *Simultaneous matching of house morphs*, *F*(1, 37) = 4.49, *p*<.05, *f* = .348. There were no further significant net effect sizes, neither on the other indicator task for object cognition speed nor on the indicator for mental speed, *F*s<1.9.

#### Summary of results of the second post-test

On the second post-test, the net effect sizes of the speed group on all indicators of speed of face cognition and of object speed were still positive though only two remained significant.

## Discussion

Face cognition is a highly important ability for social interaction. Resent research suggests that there are large individual differences in face cognition with some individuals performing extremely poorly and others extremely well. The present study investigated the possible enhancement of face cognition by training in healthy middle-aged adults with average face cognition performance. We aimed to improve specific factors of face cognition, namely the accuracy of face memory and the speed of face cognition. These factors were derived from the three-factor model proposed by Wilhelm and colleagues [5]. Two training procedures were developed. Their effects were assessed behaviorally within the training and with two post-tests. The model of face cognition established by Wilhelm et al. [5] was replicated. The training of face cognition speed had a significant and positive effect whereas no training effects were found for the memory training. The speed training led to shorter RTs on all indicators of speed, indicating lack of specificity. In the next sections, effectiveness of the training procedures and specificity of these effects for face cognition will be discussed in turn.

### Effectiveness of Training

The effectiveness of the two training procedures was tested at three different levels: first, within the trained task, second, at the manifest level with indicators that had not been trained, and third, at the latent ability level by regressing the latent factors onto treatment variables. The results of the memory training will be considered first and of the speed training second.

#### Memory Training

Despite a trend for improvement on the trained face memory task during training, there was no training-induced change at the manifest or the latent factor level. Humans practice their face cognition on a daily basis from their birth on. Therefore it might be impossible to provide a noticeable dosage of training relative to pretraining practice [27], [29], [32]. Previous studies found no training effects on face memory in healthy subjects and hence our lack of significant training results may not be all that surprising. It is possible that people with unimpaired and unaltered face memory ability already perform at the maximum of their capability as suggested before [1], [3], [5]. Face memory training might be more promising for clinical populations or in older age when face memory deficits become more severe [18], [73].

Further, the memory training task only showed a low correlation with the indicators of face memory from the test battery. This finding might indicate that the memory training task might not have tapped the same ability as have the other face memory tasks from the test battery. Another explanation for this low correlation is, that the training task was much more difficult than the other indicator tasks. The low performance of all groups on this task (compare Table 4) specifically when compared with performance on the other indicators of face memory supports this notion.

#### Speed Training

The speed group reacted faster than the other two groups on the speed training task administered at first and second post-test. Further, this group achieved significant gains in performance on all facial speed tasks in the first post-test and retained the better performance on two out of six indicators in the second post-test. Latent models were not calculated with the data of the second post-test because the unmatched control group did not participate in this test and the sample was too small for such models. The speed training group performed also faster on the speed tasks from the test battery than the other groups. The test-battery tasks in the post-test differed from the trained tasks, thus, this finding demonstrates near-transfer. At the latent level, effectiveness of the speed training was demonstrated within the pre-post-test model, the measurement model of the post-test data, and the omnibus model with pre- and post-test data. The results at the latent level are in concordance with the conclusions derived from the results for manifest variables. This suggests that the obtained results are robust to different analytic approaches.

Our speed of face cognition training procedure consisted of two very easy tasks, which targeted speed of face perception and speed of face memory respectively. RTs on both practiced tasks decreased over the course of training while at the same time the adaptation algorithm kept performance at the level of 90% correct. Shorter RTs for the odd-man-out task indicated that processes of face perception were speeded up and shorter RTs for the 1-back task indicated that face memory processes were speeded up, too. Interestingly, neither the performance on the latent factor for face perception nor for face memory was affected by this training. This result indicates that although perceptual and memory demands were incorporated into the training tasks, the demand on those factors was insufficient to cause corresponding effects on face perception or face memory. The expected effects for the speed of face cognition could be established.

These findings provide novel evidence for the plasticity of speed of face cognition. The training of processing speed is studied mainly in aging research. Our results replicate and extend earlier findings of performance enhancement through training of speed of processing in aged populations [40], [74]–[78]. In the study by Willis et al. [75] elderly participants trained processing speed. For evaluation, a performance-based functional measure of everyday speed of processing was used and showed gains in the targeted ability. Training of speed and accuracy of processing auditory information significantly enhanced processing speed [40]. Even simple retest learning enhanced the speed of processing. The first study comprised a pre-test, six retests, and a post-test and showed practice-induced performance gains in speed of processing [77]. The second study examined the persistence of these results after 8 months and still found better performance on processing speed [76]. In another study, elderly participants successfully trained on a continuous date comparison task intended to enhance their information processing speed [74].

The effect sizes for the speed of face cognition training were compared to those of training studies on speed of face processing. The effect size for the training of processing speed was *f* = .44 in the investigation of Smith et al. [40] and *f* = .15 in the study by Willis et al. [75]. For purposes of comparison to this literature, the effect sizes here are not net effects, but calculated for changes in RT when contrasting the training groups (i.e., speed vs. memory trainees). For the speed of face cognition training, the effect sizes on six indicators of speed of face cognition were medium to large (range: .35 to .98, mean: .63) and five of them were larger than those reported above. Part of these larger effect sizes might be due to the younger age of participants in the present training of the speed of face cognition as compared to the other studies [76].

Our trainings results are also comparable to two other studies that did not control for retest effects. The study by Gunther et al. [74] had no control group and administered two runs of the same Trail Making Test both in the pre- and post-test. Their effect sizes were *f* = 1.14 directly after the intervention and *f* = 1.05 for the 5-months follow-up. The retest training in the study by Yang and Krampe [76] resulted in an effect size of *f* = 2.35 after taking the same test 8 times. Therefore, these effect sizes are related to simple task specific improvements. For the speed of face cognition training discussed here, this compares best to the effect sizes for tasks practiced during the training itself: for odd-man-out task effect size was *f* = 2.22 and for the 1-back task it was *f* = 1.50. Overall, the effect sizes of our speed of face cognition training were comparable or somewhat higher than the ones reported in the literature.

The speed training group in the present study improved on speed of face cognition but scored lower on face memory in the models that controlled for individual differences at pre-test. Is it possible that subjects in that group altered their face cognition strategy to an emphasis on speed rather than accuracy At the latent level, lower scores of the speed group on the accuracy-based factors in combination with a higher score on the RT-based factor might indicate such a strategy modification. Interestingly, this pattern of face cognition performance occurred even though the instruction during training emphasized both speed and accuracy; reward points were granted and time pressure increased only when accuracy levels exceeded 90%. In models that were based only on the post-test data, the speed training did not significantly influence performance on the latent factor for face memory. Furthermore, it did not affect the scores on face perception. Thus, these results do not support the notion that the effects of the speed training are due to a shift in face processing strategy, and it deems safe to assume that at least some portion of the changes represents the intended training effects.

In summary, the speed training procedure tested here reduced RTs as intended. Training gains were evidenced at the manifest and at the latent level. The effect sizes achieved were large exceeding those of several other speed training studies for non-face stimuli. Further, near-transfer was demonstrated on independent indicators of speed of face cognition and with another kind of face stimuli. These findings parallel the literature on training speed of processing and reflect the plasticity of face processing speed for middle-aged adults.

### Specificity

Since only the training of speed of face cognition was effective, specificity will be discussed only for this training procedure. As intended, the training of speed of face cognition significantly influenced the respective latent ability factor. Further, this training generalized to a different type of face stimuli. During training, artificially generated faces were used, whereas the test battery utilized photographs of real persons. This transfer effect corresponds to the findings in other studies that also showed that training on artificial stimuli generalized to photographs [22], [79].

An analysis of specificity comparing training effects to face and to object stimuli revealed that our training procedure also enhanced speed of object cognition as indicated by significant improvement of performance on the factor for object speed. Further exploring this finding and incorporating the two speed factors into one implied that there is no need for such a distinction. This result corresponds well to the finding that already for the pre-test data such a differentiation was not sustainable. Wilhelm et al. [5] found face processing speed to be strongly correlated with mental speed but nonetheless a distinct ability. Two further studies that explored the specificity of speed of face cognition reported even stronger correlations between their specified speed factors with stimuli from different domains. One study found a perfect correlation between the speed of face cognition and the speed of processing for emotional expressions, however the speed of face cognition factor correlated only moderately with object cognition speed [80]. More importantly, the other study showed that the speed measures of face cognition reflect the same ability as speed measures for objects [81]. The authors concluded that this ability captures the speed of processing complex visual stimuli. The present study confirms and extends these findings by demonstrating that training aimed at speed of face cognition also enhances performance for processing non-face stimuli. These findings suggest that the training program used here affected mechanisms important for fast responses to complex stimuli but that are not specific to face cognition. Indeed, it would be very interesting to find out whether speed of face cognition training also enhances the speed of processing for emotional expressions, whether it extends to measures of daily life or professional success, and what the upper limits for improvement are.

Some studies of cognitive aging also demonstrated that training processing speed transfers to other cognitive abilities. Ball, Edwards, and Ross [78] re-analyzed the data from six studies on training speed of processing with older adults. They found that training-induced improvements transferred to everyday abilities as well as to driving performance. In the study by Gunther et al. [74], a broadly aimed computer-assisted training improved information processing speed in older adults and also improved learning of verbal material and reduced interference tendency, a cause of memory loss. Training on speed and accuracy of processing auditory information improved the targeted abilities and transferred to untrained standardized measures of memory and attention [40]. Most interestingly, trainees' self-reports suggested that the training gains may be behaviorally significant. In the latter two studies, trainees practiced on sets of broad-based exercises, therefore, the transfer of improvements cannot be directly attributed to the speed of processing training. However, other studies found no transfer of speed of processing training to activities of daily living [75], [82]. The authors of the latter studies suggest that the advantaged nature of their samples might have caused ceiling effects on measures of everyday cognitive abilities and, thus, left no room for improvement through training.

Summarizing, cognitive aging research indicates that speed of processing may transfer to measures of everyday functioning specifically for persons with declining speed of processing. The effects of speed of face cognition training investigated in our study improved speed for face cognition, speed for object cognition, and also enhanced performance on a mental speed task with letters as stimuli. Therefore, the indication of this training was reassessed and extended to the ability of perceiving and recognizing complex stimuli swiftly. Remarkably, the effects of our training procedure applied to a young to middle-aged sample of participants were comparatively large and obtained in a tightly controlled study design. It remains for future research to explore the transfer effects from the training developed here to measures of everyday functioning. If this intervention can help enhancing the speed of processing in general or prevent it from age-related decline, there are obvious practical applications for such training.

### Conclusions

The methodological and technical approach of this study was sophisticated concerning concept, realization, and data analysis as compared with previous studies on training face cognition [21], [27], [32], [33]. However, the training of face memory was not effective. Having full knowledge of previous face cognition training procedures and their results (significant and not significant ones) will help future researchers to identify the mechanisms for plasticity and understand their limitations.

Training speed of face cognition significantly enhanced the targeted ability, the effects were large, and persisted over time. The effects were present for the training tasks as well as at the latent ability level in pre-post comparisons. Speed training for faces also enhanced the speed of object cognition and mental speed. In line with previous research, the effects generalized to other speed indicators substantiating far-transfer [78], [80]. Given the lack of distinctiveness of the speed of face cognition the indication for the training needs to be reconsidered: Apparently, the intervention affected the speed of processing complex visual stimuli.

This is the first study to show that training speed of face cognition with an unsupervised, computer-based intervention can improve the targeted ability and extend to gains in speed for perception and recognition of complex stimuli. These results were obtained in a middle-aged sample of normal, unimpaired participants. Because speed of processing is an influential cognitive ability for independent everyday functioning of elderly persons [75], [78], [83], [84], such an easy to administer, cost-efficient program might be well-suited to make training benefits more widely accessible also to the general public.

## Supporting Information

File S1 **This online supplement provides a brief description of all tasks included in the post-tests.**
(DOCX)
