# Supplementary material for: Can Training Enhance Face Cognition Abilities in Middle-Aged Adults?
Source: PLoS One. 2014 Mar 14;9(3):e90249. doi: 10.1371/journal.pone.0090249 (PMC3954557; doi:10.1371/journal.pone.0090249)
Supplement: File S1 — This online supplement provides a brief description of all tasks included in the post-tests. (DOCX) [file pone.0090249.s001.docx]

**Supplement**

Can Training Enhance Face Cognition Abilities?

Dominika Dolzycka, Grit Herzman, Werner Sommer, Oliver Wilhelm

A brief description of all indicators used in the study is provided. For more detailed information see Hildebrandt et al. (2010, 2011).

*Facial resemblance (FP1)*

A target face was presented in three-quarter view in the upper half of the screen together with two morphs in frontal view in the lower half of the screen. Morphs were created by combining the target and a second face to different degrees (e.g., morph 1: 20% target and 80% face B, morph 2: 40% target and 60% face B). For a total of 48 trials, participants identified the morph that was most similar to the target.

*Sequential matching of part-whole faces – condition part (FP2) and condition whole (FP3)*

A target face was briefly shown. Subsequently, a facial feature from the target (eyes, nose, or mouth) was presented together with the same feature taken from a different face (part condition, FP 2). Participants identified which feature belonged to the target face. In the whole condition (FP 3), two whole faces were shown: one was the target, the other one was a distracter composed of the target face with one facial feature taken from a different face. Targets had to be identified. There were 30 trials each for the part and the whole condition.

*Simultaneous matching of spatially manipulated faces – condition upright (FP4) and condition inverted (FP 5)*

Two pictures of a face were presented at the same time. Both pictures were either upright (FP4, 30 trials) or turned upside down (inverted, FP5, 30 trials). Half of the trials presented the exact same face in both pictures. The other half presented spatially manipulated faces in which one spatial relationship between the facial features (e.g. between eyes and nose, between eyes, between mouth and nose) was manipulated in one of the faces. Participants identified whether pictures were identical or not.

*Acquisition curve (FM1)*

Fifteen faces had to be memorized as well as possible within 45 seconds (study phase). An unrelated task (2 min) intervened between study and recognition phase. The recognition phase had five runs. All studied faces appeared in each run, every time combined with new distracters. Participants identified the studied faces with feedback which showed the target to ensure further learning. The whole task was repeated with 15 new targets for a total of 30 target faces. Performance was measured as mean accuracy across both parts and all recognition blocks.

*Decay rate of learned faces (FM2)*

Recognition performance of the 30 faces learned during FM 1 was assessed in the same session approximately 2.5 hours after administration of FM 1. Studied faces were shown together with new distracters. Participants identified the studied faces.

*Eyewitness testimony (FM3)*

Two faces were shown, one of which had been presented in two previous tasks. Participants indicated the previously seen faces for 46 trials in this incidental memory task.

*Recognition speed of learned faces (SFC1)*

The task had four parts. A study phase was followed by a recognition phase after a 4-minute delay. Four faces were studied for one minute. During the delay, participants completed a reasoning test (GA3). During recognition, four studied and four new faces were shown one at a time. Participants indicated whether the shown face was studied or new. There were a total of 32 trials.

*Delayed non-matching to sample (SFC2)*

A target face was presented for one second, followed by a delay of 4 seconds. Then, the target face was shown combined with a new face. The new face had to be identified for a total of 46 trials.

*Simultaneous matching of faces from different viewpoints (SFC3)*

In each of 30 trials, two faces were shown. They were presented in the diagonal of the screen, one in frontal and the other in three-quarter view. Participants identified whether the faces depicted the same person or different persons.

*Simultaneous matching of upper face-halves – condition aligned (SFC4) and condition non-aligned (SFC5)*

Faces were divided horizontally into upper and lower halves. The upper half of a given face was added to the lower half of a different face, creating a new composite face. Upper and lower halves were always taken from different persons. In SFC4 (30 trials), face halves were aligned to form a new face. In SFC5 (30 trials) the face halves were not aligned instead the right or left face-corners of the top face were positioned above the nose of the bottom face. In each trial, two composite faces were shown in the diagonal of the screen. Participants decided whether the upper face-halves showed the same face (50% of trials) or different faces.

*Simultaneous matching of morphs (SFC6)*

Faces for each trial were created by morphing the same two faces to varying degrees. Pairs of morphs were either similar or dissimilar (50% of trials). Participants made a similarity decision for each of 30 trials.

*Sequential matching of part-whole houses – condition part (OC1) and condition whole (OC2)*

The task was procedurally identical to FP2 and FP3 but used houses as stimuli. Instead of facial features, house features (window, door, or roof) were used.

*Delayed non-matching to sample of houses (OC3)*

The task was procedurally identical to SFC2 but used houses as stimuli.

*Simultaneous matching of houses (OC4)*

The task was procedurally identical to SFC6 but used houses as stimuli. Instead of morphing houses, unaltered pictures were used.

*Immediate (GA1) and delayed memory (GA2)*

These tasks were adapted from IDM3 (GA1) and IDM4 (GA2) of the Wechsler Memory Scale (Härting, Markowitsch, Neufeld, Calabrese, & Deisinger, 2000). Tasks required studying eight first and last names. There were three study phases. All eight names were shown in each study phase. Immediately following each study phase, participants had to recall the last name when the first name was given (GA1). After a delay of 1.5 hours (GA2), participants had to recall the last names again. Responses were typed using the keyboard.

*General cognitive ability (GA3)*

Sixteen items from the original test by Raven, Court, and Raven (1979) were used. Each trial showed a three by three matrix of symbols, with one symbol missing at the bottom right. Participants identified the symbol that logically completed the matrix out of eight different options.

*Mental speed (GA4)*

The “Finding As” task (Danthiir, Wilhelm, & Schacht, 2005) was used. Each of 80 trials showed one German word. Participants identified whether the shown word contained the letter “A” or not (50% of the trials).

**References**

Danthiir, V., Wilhelm, O., & Schacht, A. (2005). Decision speed in intelligence tasks: correctly an ability? *Psychology Science, 47*, 200-229.

Härting, C., Markowitsch, H. J., Neufeld, H., Calabrese, P., & Deisinger, K. (2000). *Die Wechsler-Memory-Scale-Revised: Deutschsprachige adaptation [The Wechsler Memory Scale revised: German adaptation]*. Bern, Switzerland: Huber.

Hildebrandt, A., Sommer, W., Herzmann, G., & Wilhelm, O. (2010). Structural invariance and age-related performance differences in face cognition. *Psychology and Aging, 25*, 794-810.

Hildebrandt, A., Wilhelm, O., Schmiedek, F., Herzmann, G., & Sommer, W. (2011). On the specificity of face cognition compared to general cognitive functioning across adult age. *Psychology and Aging, 26*, 701-715.

Raven, J. C., Court, J. H., & Raven, J. (1979). *Manual for Raven’s Progressive Matrices and Vocabulary Scales.* London, England: Lewis.
